# Predicting the geographical distribution and niche characteristics of *Cotoneaster multiflorus* based on future climate change

**DOI:** 10.3389/fpls.2024.1360190

**Published:** 2024-05-08

**Authors:** Qiuliang Huang, Haoyang Liu, Changshun Li, Xiaoru Zhu, Zongsheng Yuan, Jialiang Lai, Minghui Cao, Zhenbei Huang, Yushan Yang, Shenglan Zhuo, Zengwei Lü, Guofang Zhang

**Affiliations:** ^1^ College of Forestry, Fujian Agriculture and Forestry University, Fuzhou, Fujian, China; ^2^ Service Center, Fujian Meteorological Bureau, Fuzhou, Fujian, China; ^3^ Project Department, Norite International Construction Group Co., Xi’an, Shaanxi, China; ^4^ Institute of Oceanography, Minjiang University, Fuzhou, Fujian, China

**Keywords:** *Cotoneaster multiflorus*, MaxEnt 2.0 model, distribution, niche, arid and semi-arid areas, ecological restoration

## Abstract

**Introduction:**

Arid and semi-arid regions are climate-sensitive areas, which account for about 40% of the world’s land surface area. Future environment change will impact the environment of these area, resulting in a sharp expansion of arid and semi-arid regions. *Cotoneaster multiflorus* is a multi-functional tree species with extreme cold, drought and barren resistance, as well as ornamental and medicinal functions. It was found to be one of the most important tree species for ecological restoration in arid and semi-arid areas. However, bioclimatic factors play an important role in the growth, development and distribution of plants. Therefore, exploring the response pattern and ecological adaptability of *C. multiflorus* to future climate change is important for the long-term ecological restoration of *C. multiflorus* in arid and semi-arid areas.

**Methods:**

In this study, we predicted the potential distribution of *C. multiflorus* in China under different climate scenarios based on the MaxEnt 2.0 model, and discussed its adaptability and the major factors affecting its geographical distribution.

**Results:**

The major factors that explained the geographical distribution of *C. multiflorus* were Annual precipitation (Bio12), Min air temperature of the coldest month (Bio6), and Mean air temperature of the coldest quarter (Bio11). However, *C. multiflorus* could thrive in environments where Annual precipitation (Bio12) >150 mm, Min air temperature of the coldest month (Bio6) > -42.5°C, and Mean air temperature of the coldest quarter (Bio11) > -20°C, showcasing its characteristics of cold and drought tolerance. Under different future climate scenarios, the total suitable area for *C. multiflorus* ranged from 411.199×10^4^ km² to 470.191×10^4^ km², which was 0.8~6.14 percentage points higher than the current total suitable area. Additionally, it would further shift towards higher latitude.

**Discussion:**

The MaxEnt 2.0 model predicted the potential distribution pattern of *C. multiflorus* in the context of future climate change, and identified its ecological adaptability and the main climatic factors affecting its distribution. This study provides an important theoretical basis for natural vegetation restoration in arid and semi-arid areas.

## 1 Introduction

Since the last interglacial period, multiple global climate fluctuations have profoundly affected the vegetation and ice cover volume in most regions on the world (Zhang et al., 2019). About 40% of the world’s land surface is arid or semi-arid, and with the continued warming in the second half of the 20th century, most parts of the world are experiencing a trend towards drought (Dai, 2011; Haile et al., 2019; Lan et al., 2022). The Millennium Drought in Australia from 2002 to 2009 (Van Dijk et al., 2013), the summer drought in Europe in 2003 (Ciais et al., 2005), the East African drought in 2011 (Lott et al., 2013), and the Mega-Drought in the Southwest United States from 2010 to 2012 (Gleick and Heberger, 2012) were examples for this trend. China is one of the countries with a large area of drought and water scarcity, and it faces serious water resource shortages. The arid and semi-arid areas of China cover a total area of 5.66 million square kilometers, accounting for about 58.6% of the country’s land area, mainly distributed in North China, Northwest China, Inner Mongolia, the Loess Plateau, and the most parts of Qinghai-Tibet Plateau (Yan and Gao, 2003).

It is crucial to select appropriate tree species and establish long-term ecological restoration plans for drought-prone and semi-arid regions by utilizing species distribution data and environmental information to generate niche-based models that explore and predict species’ response patterns to future climate change (Zhang et al., 2020; Subedi et al., 2023, 2024; Varol et al., 2022). The Maximum Entropy (Maxent) model is a density estimation and species distribution model (Phillips et al., 2006) that is one of the most effective and widely used methods for studying the impact of climate change on species habitat suitability (Araújo et al., 2019). It has high prediction accuracy, good generality, strong stability, and performs well with small sample sizes compared to other modeling methods (Elith et al., 2006; Khanum et al., 2013; Ji et al., 2020). The Maxent model can also provide an intuitive representation of species distribution areas at different time periods. In contrast, it is possible to identify the response patterns of the same species to different climate changes (Shi et al., 2022). The Maxent model has been widely used for species distribution modeling on Earth (Jiang et al., 2022; Huang et al., 2023; Lai et al., 2023; Zhao et al., 2023).

The concept of niche has been used to explain the spatial and temporal distribution, abundance, and resource utilization of species (Chase and Leibold, 2003; Peterson et al., 2011). The differences of niche among species determine the potential fundamental drivers of species coexistence and ecosystem functionality (Godoy et al., 2020). The two main characteristics of niche are niche breadth and niche overlap referred to species in community. Niche breadth measures the population’s ability to utilize resources, while niche overlap reflects the degree of similarity in the competition for environmental resources among different populations, providing references for community stability (Colwell and Futuyma, 1971; Gu et al., 2017; Hu et al., 2022). Therefore, in order to fully investigate the importance of plant-plant interactions in a changing environment, it is necessary to conduct field surveys and study based on niche, which helps us better understand species interactions, distribution, coexistence, and the underlying mechanisms, and provides theoretical basis for forest management and natural vegetation restoration.

The *Cotoneaster* genus, a relatively ancient and extensive genus with over 90 species, belongs to the Rosaceae family. It is mainly distributed in Asia and Europe, with more than 50 species found in China, most of which are native to the region (Monier et al., 1998; Chang and Jeon, 2003; Khan et al., 2009). The diversification of the *Cotoneaster* genus can be traced back to the early Miocene period, approximately 20 million years ago, and all existing species have evolved since the middle Miocene, around the same time (Yang et al., 2022). *Cotoneaster* species are notable for their widespread occurrence of apomixis, interspecific hybridization, and intraspecific morphological variation (Bartish et al., 2001; Meng et al., 2021). Among these, *C. multiflorus* is a shrub that belongs to the Maloideae subfamily of the Rosaceae family (Yang et al., 2022).

**Figure 1:**
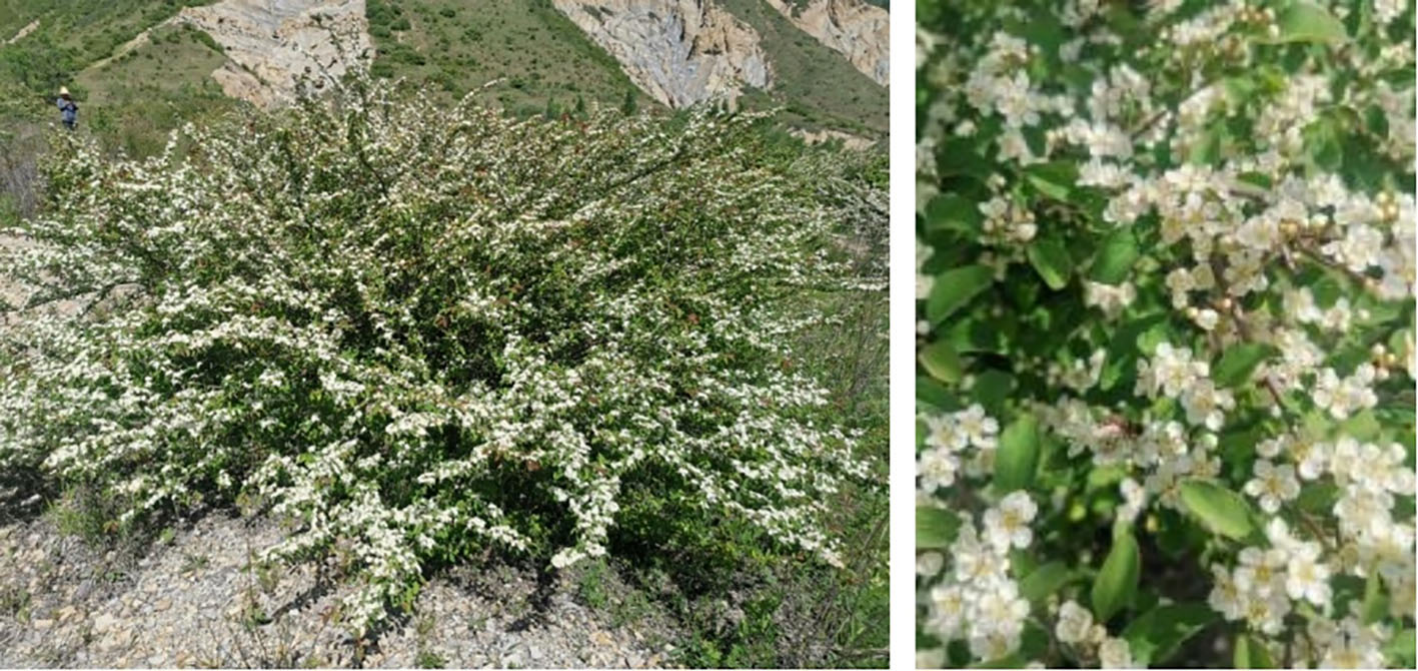
*Cotoneaster multiflorus* growing on bedrock.

*C. multiflorus* is a multi-functional tree species that has not been fully explored. For example, numerous *Cotoneaster* species have gained popularity in garden landscapes due to their attractive leaves, colorful flowers, and vibrant fruits (Bartish et al., 2001; Liu X. P. et al., 2018). Besides, various types of *Cotoneaster* species are used to treat bronchitis, gastritis, vasculitis, wound infections, and as natural antioxidants (Palme et al., 1994; Khan et al., 2009; Liu R. H. et al., 2018). Moreover, with its extensive root system and tolerance to cold, drought, and poor soil, *C. multiflorus* serves as a pioneering tree species for vegetation restoration in arid and semi-arid loess hilly areas and rocky mountainous ecological transition zones (Kou, 2016) (**Figure 1**). However, evidence on *C. multiflorus* is still largely lacking, focusing mainly on breeding (Yu et al., 2018; Sun et al., 2020), introduction trials (Chen et al., 2021), extraction of constituents (Chang and Jeon, 2003; Jia, 2022), molecular-level classification (Yang et al., 2022), and morphological studies (Ding et al., 2007; Niaki et al., 2019). Also, much less is known about the current distribution, future response to global climate change, community structure, and ecological adaptation of *C. multiflorus*. Therefore, we conducted field investigations and employed the ENMeval data package to assist in selecting the MaxEnt model. The study aimed at predicting the potential distribution of *C. multiflorus* under the context of climate change, and identity the primary climatic factors that restrict its distribution, as well as its ecological adaptability. This study will provide novel evidence for developing effective policy for ecological restoration in arid and semi-arid areas.

## 2 Materials and methods

### 2.1 The study area

**Figure 2:**
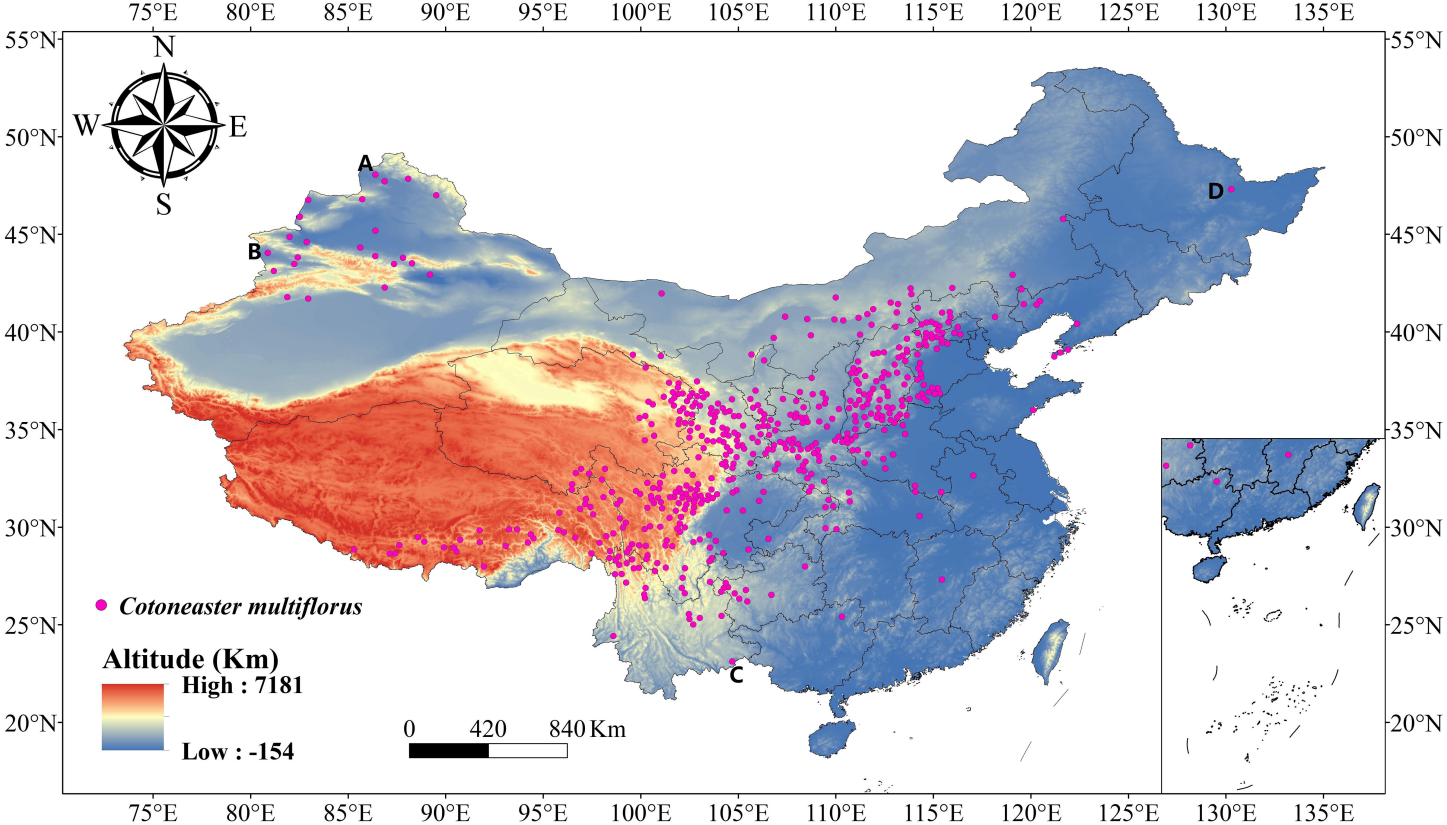
The distribution of *C. multiflorus*. **(A)** The northern part of Altay Prefecture in Xinjiang Uygur Autonomous Region; **(B)** The western end of Bortala Mongolian Autonomous Prefecture, Xinjiang Uygur Autonomous Region; **(C)** The southwestern part of Yunnan Province; **(D)** The western end of Wanda Mountains in eastern Heilongjiang Province.

According to “Flora of China”, *C. multiflorus* is widely distributed in China, with its natural range extending from the northern part of Altay Prefecture in Xinjiang Uygur Autonomous Region to the southwestern part of Yunnan Province, and from the western end of Wanda Mountains in the eastern part of Heilongjiang Province to the western end of Bortala Mongolian Autonomous Prefecture, Xinjiang Uygur Autonomous Region. The natural distribution range of *C. multiflorus* is approximately 80°~130° E and 23°~48° N. To avoid overlooking potential suitable areas for *C. multiflorus* within China and to provide reference for future seed introduction, the study area was expanded to 72°~136° E and 0°~55° N (**Figure 2**).

### 2.2 Data on the distribution of *C. multiflorus*

The distribution data of *C. multiflorus* in this study were obtained from field surveys and databases. We conducted field surveys of natural populations of *C. multiflorus* in the Ningxia Hui Autonomous Region from March to July 2021, collecting 84 distribution records. Other distribution records were obtained by searching specimen and literature databases, including Global Biodiversity Information Facility (GBIF)^1^, National Specimen Infrastructure (NSII)^2^, Chinese Virtual Herbarium (CVH)^3^, Plant Species Information System^4^, China National Knowledge Infrastructure (CNKI) and Web of Science (SCI), as well as native flora, collecting 2,057 distribution records. A total of 2,141 distribution records were obtained in this study. To reduce model errors caused by clustering effects, we imported the *C. multiflorus* distribution point data into ArcGIS, removed artificially planted distribution points, ambiguous records, duplicate distribution points, and conducted buffer zone analysis (Li et al., 2016). Only one distribution point was retained within each 1 km × 1 km grid, resulting in a final set of 539 valid samples.

### 2.3 Data on environmental variables

The provincial administrative map of China used in this study was sourced from the National Basic Geographic Information System Database of China. Contemporary and future climate data [2050s (2041-2060), 2090s (2081-2100)] were obtained using BCC-CMN2-MR in Worldclim 2.1, including SSP1-RCP2.6 (SSP126), SSP2-RCP4.5 (SSP245), and SSP5-RCP8.5 (SSP585). ‘SSP’ represents Shared Socioeconomic Pathway, with SSP126, SSP245, and SSP585 representing low, middle, and high greenhouse gas emission scenarios respectively, while ‘RCP’ represents Representative Concentration Pathways (Popp et al., 2016). This study included seven climate backgrounds (Current, SSP126-2050s, SSP126-2090s, SSP245-2050s, SSP245-2090s, SSP585-2050s, SSP585-2090s), each containing 19 environmental variables with a spatial resolution of 2.5’. To avoid overfitting of the model, Pearson correlation analysis was used in SPSS 25.0 software to screen environmental variables. Environmental variables with |*r*|< 0.8 were retained, while those with |*r*| > 0.8 were selected based on their close relationship with *C. multiflorus* growth (Tang et al., 2021; Xu D. et al., 2019). Finally, 8 environmental variables were selected, as shown in **Table 1**.

**Table 1:** Filtered environmental variables.

Data type	Variable	Specific name	Unit
Climatic factor	Bio6	Min air temperature of the coldest month	°C
	Bio8	Mean air temperature of the wettest quarter	°C
	Bio9	Mean air temperature of the driest quarter	°C
	Bio11	Mean air temperature of the coldest quarter	°C
	Bio12	Annual precipitation	mm
	Bio14	Precipitation of the driest month	mm
	Bio15	Precipitation of the driest month	mm
	Bio19	Precipitation of the coldest quarter	mm

### 2.4 Model specification and accuracy evaluation

When optimizing the MaxEnt model by calling the ENMeval package in R software, adjusting the optimal values of the regularization multiplier (RM) and feature categories (FC) can significantly improve the prediction accuracy (Phillips and Dudík, 2008; Cobos et al., 2019; Kass et al., 2021). In this study, we used the EMNeval 2.0 program package in R v4.3.1 for parameter optimization, setting RM in the range of 0.1 to 6 with an interval of 0.5, resulting in a total of 13 regulation multipliers. We also used 10 FC: H, L, LQ, LQH, LQHP, and LQHPT, where L represents linear, Q represents quadratic, H represents hinge, P represents product, and T represents threshold. The ENMeval program package tested the above 78 parameter combinations, and the model fitting degree and complexity were determined using the delta AICc model based on the Akaike information criterion (Kuiper and Hoijtink, 2011). After determining the RM and FC parameter combinations, the collected *C. multiflorus* sample distribution points and environmental variables were imported into the MaxEnt software. 75% of the sample data was randomly selected as the training set, and 25% as the test set. The maximum number of iterations was set to 1000, and the performance of Bootstrap was repeated 10 times. Other MaxEnt parameters were set to default values.

The simulation accuracy was evaluated using the Receiver Operating Characteristic curve (ROC curve). The area enclosed by the ROC curve and the horizontal axis is the AUC (Area Under Curve) value. The range of AUC values is 0~1, with higher values indicating better prediction performance. Generally speaking, an AUC value less than 0.7 indicates poor prediction performance, 0.7~0.8 indicates relatively accurate, 0.8~0.9 very accurate, and 0.9~1 extremely accurate (Swets, 1988; Araújo et al., 2005; Mahmoodi et al., 2022).

### 2.5 Classification of suitable areas for *C. multiflorus*

The simulation result files were imported into ArcGIS software for classification of suitability areas. This study employed Jenks’ natural breaks grading method to divide the habitat of *C. multiflorus* into four levels: unsuitable area (0~0.1), low suitable area (0.1~0.3), medium suitable area (0.3~0.5), and highly suitable area (0.5~1) (Ren et al., 2020). Furthermore, using the ‘Overlay Analysis’ tool in ArcGIS 10.5, the contemporary suitable habitat of *C. multiflorus* was served as a reference to compare and define the following three scenarios based on changes in the range of *C. multiflorus*: (1) Retained suitable area: areas suitable in both contemporary and future periods; (2) Increased suitable area: areas currently unsuitable but suitable in the future; (3) Lost suitable area: areas currently suitable but unsuitable in the future. Area statistics and visual representation were conducted using ArcGIS 10.5 (Khwarahm, 2020).

### 2.6 Calculation of niche breadth and niche overlap

Importance value is a quantitative index used to characterize the status of species in the community. Great importance value means the species is dominant in the community (Feroz et al., 2008). After field investigation (**supplementary 1**), the main tree species with important value greater than 1% were selected for niche research. Niche overlap reflects the similarity in resource utilization among plants. A higher niche overlap index suggests greater similarity in resource requirements and increased competition (Yuan et al., 2021). Niche breadth was measured by Levins index (*B_L_*) and Shannon index (*B_S_*), niche overlap was measured by Pianka index (*O_ik_*). The calculation formulas were as follows in Equations 1–4 (Fang et al., 2009; Liu R. H. et al., 2018; Liu et al., 2006):


(1) Importance value=(relative frequency+relative dominance) /3

(2) $$B_{L}=1/\sum_{j=1}^{r}\;p_{ij}^{2}$$

(3) $$B_{S}=-\sum_{j=1}^{r}\;p_{ij}\text{ In }p_{ij}$$

(4) $$O_{ik}=\sum_{j=1}^{r}\;p_{ij}\;p_{kj}/\sqrt{\sum_{j=1}^{r}\;p_{ij}^{2}\;\sum_{j=1}^{r}\;p_{kj}^{2}}$$

In the above formulas, *P_ij_* is the ratio of utilization of species *i* on resource position *j* to the total utilization of all resource positions, *P_ik_* is in the same way. *r* represents the total number of resource bits. The value domains of *B_L_* and *B_S_* are [1,*r*] and [0,1nr], respectively. *O_ik_* is the niche overlap index between species *i* and *k*, ranging from 0 to 1.

## 3 Results

### 3.1 Model accuracy evaluation

**Figure 3:**
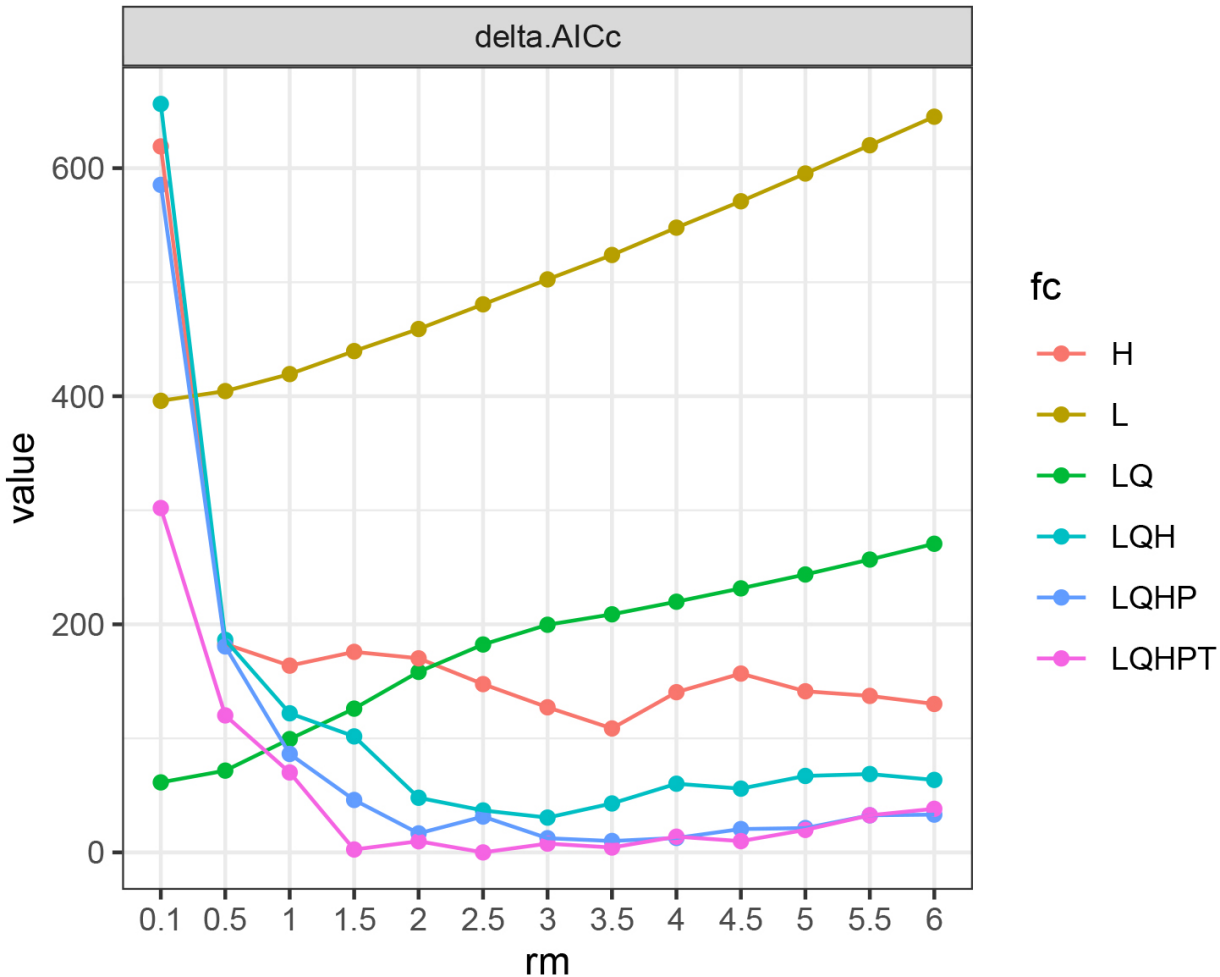
AICc value of parameter combinations based on the ENMeval calculation. fc, feature categories; rm, regularization multiplier; AICc, Akaike information criterion correction; L, linear; Q, quadratic; H, hinge; P, product; T, threshold. H, L, LQ, LQH, LQHP and LQHPT mean different feature categories. ΔAICc = 0 means the model with this parameter combination is the optimal one.

**Figure 4:**
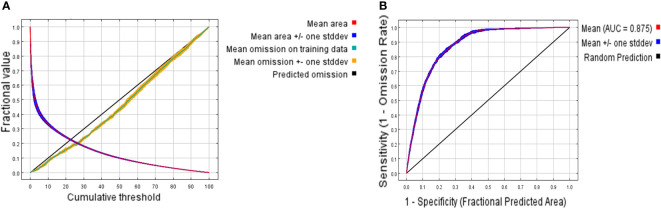
ROC curves of MaxEnt result. **(A)** The ROC verification curve of Maxent model; **(B)** Jackknife test of the importance of variables. The area enclosed by ROC curve and x axis is AUC (Area Under Curve) value. High AUC values indicate better prediction performance.

When FC=LQHPT and RM=2.5, the ΔAICc value reached its minimum (ΔAICc = 0) (**Figure 3**), indicating that the model with this parameter combination was the optimal one. Under these parameters, the result of MaxEnt prediction showed that the value of AUC was 0.875 (**Figure 4**).

### 3.2 Analysis of environmental variables affecting potential distribution of *C. multiflorus*

According to **Table 2**, the top five contributing environmental variables were Annual precipitation (Bio12, 32.9%), Mean air temperature of the coldest quarter (Bio11, 25.0%), Min air temperature of the coldest month (Bio6, 13.8%), Precipitation of the coldest quarter (Bio19, 11.4%), and Precipitation of the driest month (Bio14, 6.2%), totaling 89.3%. The permutation importance ranked the top five environmental variables as Annual precipitation (Bio12, 37.6%), Mean air temperature of the coldest quarter (Bio11, 20.0%), Precipitation of the coldest quarter (Bio19, 14.4%), Precipitation of the driest month (Bio14, 11.0%), and Min air temperature of the coldest month (Bio6, 8.1%), totaling 91.1%.

**Table 2:** Various parameters of the main environmental variables of *C*. *multiflorus*.

Environmental variables	PC(%)	PI(%)	RTGw	RTGo	TGw	TGo	AUCw	AUCo
Annual precipitation (Bio12)	32.9	37.6	0.8281	0.3783	0.8725	0.4259	0.8459	0.7446
Mean air temperature of the coldest quarter (Bio11)	25.0	20	0.941	0.4495	0.9899	0.4686	0.8633	0.7586
Min air temperature of the coldest month (Bio6)	13.8	8.1	0.9422	0.4676	0.9962	0.4878	0.8642	0.765
Precipitation of the coldest quarter (Bio19)	11.4	14.4	0.9403	0.2331	0.9907	0.252	0.8633	0.6849
Precipitation of the driest month (Bio14)	6.2	11.0	0.9432	0.1692	0.9897	0.1945	0.8631	0.661
Mean air temperature of the wettest quarter (Bio8)	5.4	2.6	0.9322	0.1797	0.9733	0.2247	0.8606	0.6865
Mean air temperature of the driest quarter (Bio9)	3.5	0.3	0.9472	0.3987	0.9937	0.4208	0.8637	0.7452
Precipitation of the driest month (Bio15)	1.8	6.0	0.9135	0.0887	0.9541	0.1167	0.8582	0.6354

PC is Percent contribution; PI is Permutation importance; RTGw is the regularized training gain without using the variable; RTGo is the regularized training gain using the only variable; TGw is the test gain without using the variable; TG_O_ is the test gain using the only variable; AUCw is the area under the receiver operating characteristic curve without using the variable; AUCo is the area under the working characteristic curve of the subjects using the only variable.

**Figure 5:**
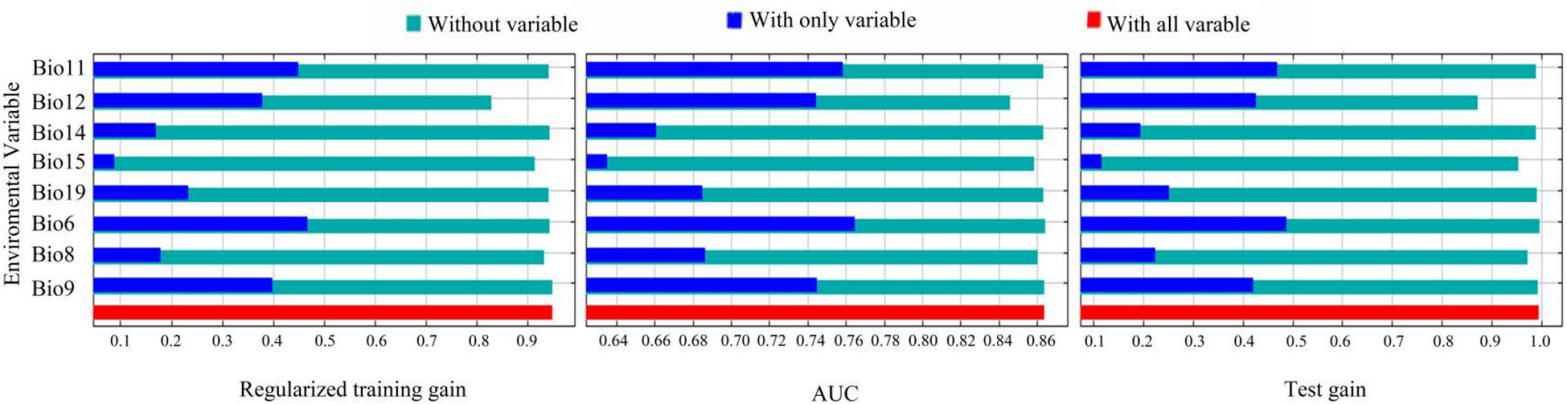
The environmental variables that have the most impact on *C. multiflorus*. Bio11: Mean air temperature of the coldest quarter; Bio12: Annual precipitation; Bio14: Precipitation of the driest month; Bio15: Precipitation of the driest month; Bio19: Precipitation of the coldest quarter; Bio6: Min air temperature of the coldest month; Bio8: Mean air temperature of the wettest quarter; Bio9: Mean air temperature of the driest quarter.

The Jackknife test (**Figure 5**) showed that when individual environmental variables were sequentially excluded, the model’s regularized training gain, test gain, and AUC values decreased most notably for Annual precipitation (Bio12), Precipitation of the driest month (Bio15), and Mean air temperature of the wettest quarter (Bio8). When using only individual variables, the top three with the highest regularized training gain and AUC value gains included Min air temperature of the coldest month (Bio6), Mean air temperature of the coldest quarter (Bio11), and Mean air temperature of driest quarter (Bio9), while the top three with the highest test gain included Min air temperature of the coldest month (Bio6), Mean air temperature of the coldest quarter (Bio11), and Annual precipitation (Bio12).

**Figure 6:**
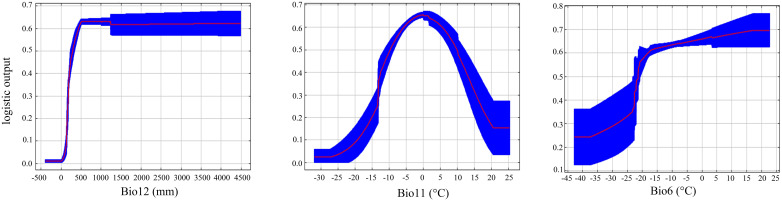
Suitable range of *C. multiflorus* for environmental variables. Bio11: Mean air temperature of the coldest quarter; Bio12: Annual precipitation; Bio14: Precipitation of the driest month; Bio15: Precipitation seasonality; Bio19: Precipitation of the coldest quarter; Bio6: Min air temperature of the coldest month; Bio8: Mean air temperature of the wettest quarter; Bio9: Mean air temperature of the driest quarter.

The response curves of main environmental variables (**Figure 6**) indicated that *C. multiflorus* could thrive in environments with Annual precipitation (Bio12) greater than 150 mm, Min air temperature of the coldest month (Bio6) higher than -42.5°C, and Mean air temperature of the coldest quarter (Bio11) higher than -20°C.

### 3.3 Geographical distribution of *C. multiflorus* under different climate scenarios

**Figure 7:**
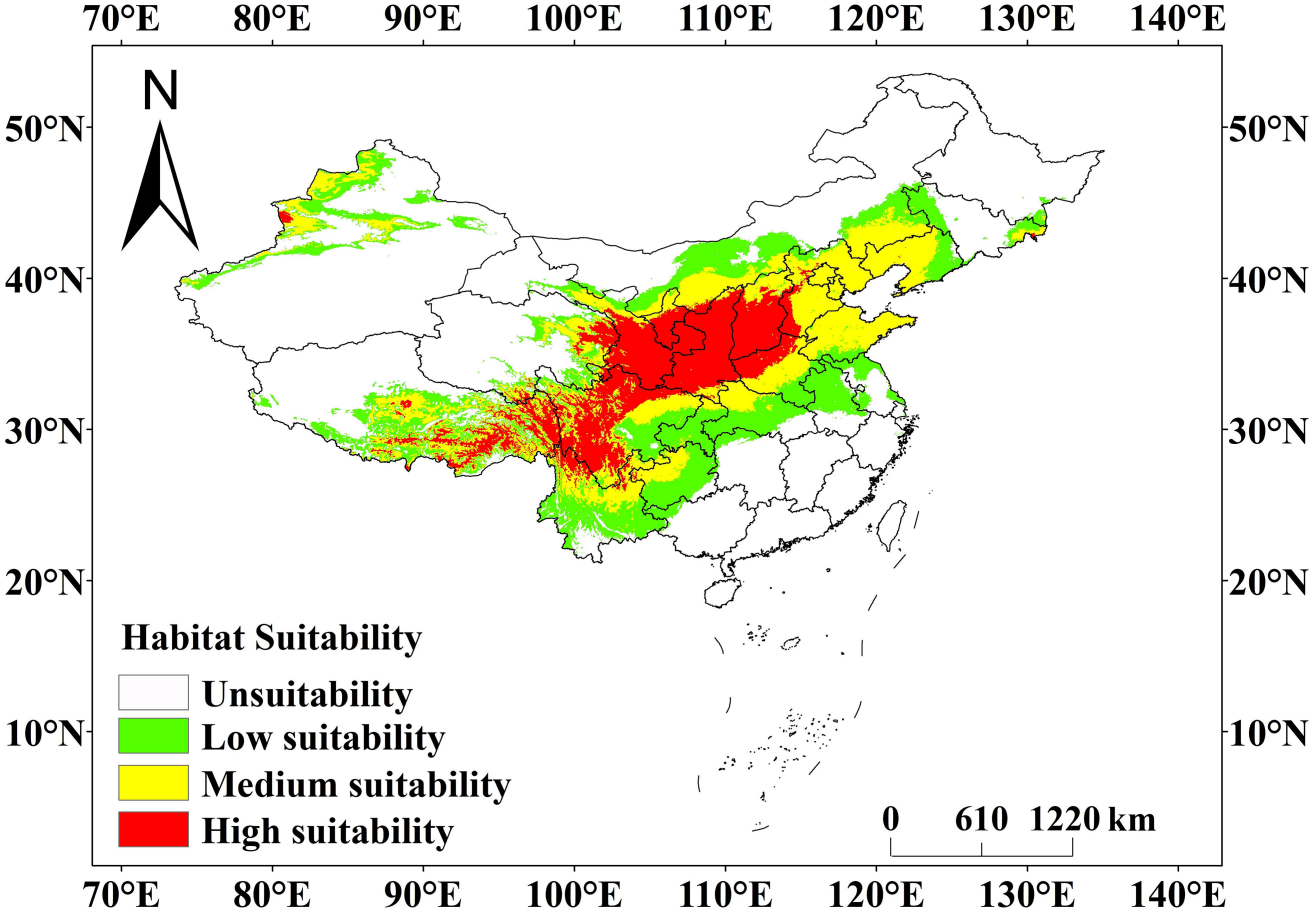
Prediction of current geographical distribution pattern of *C*. *multiflorus* by MaxEnt model. This study employed Jenks’ natural breaks grading method to divide the habitat of *C. multiflorus* into four levels: unsuitable area (0~0.1), low suitable area (0.1~0.3), medium suitable area (0.3~0.5), and highly suitable area (0.5~1).

The prediction results of the MaxEnt model (**Figure 7**) showed that the current potential suitable area of *C. multiflorus* was much larger than the actual collection distribution range (**Figures 1**, **7**). The total suitable area of *C. multiflorus* during this period was 411.20 × 10^4^ km^2^, accounting for 42.83% of the total area of the country (**Table 3**). The highly suitable areas were mainly distributed in a few areas of Shaanxi, Shanxi, southern Ningxia, southern Gansu, northern Sichuan, Xinjiang Uygur Autonomous Region, and Tibet Autonomous Region, concentrated in arid, semi-arid, and semi-humid areas on both sides of the isohyet of 400 mm (**Figure 7**), indicating that *C. multiflorus* had strong adaptability and a wide distribution. The highly, medium and low suitable areas accounted for 24.82%, 36.19% and 38.99% of the total suitable area, respectively.

**Table 3:** The change of suitable areas of *C. multiflorus* in different periods (unit: ×10^4^ km^2^).

Period	Current	SSP126		SSP245		SSP585	
		2050s	2090s	2050s	2090s	2050s	2090s
Unsuitable area	548.80	509.82	536.11	537.43	541.09	536.29	489.81
Low suitable area	160.32	165.21	174.50	168.03	169.51	164.36	170.72
Medium suitable area	148.82	118.05	146.27	145.21	149.31	151.36	130.46
Highly suitable area	102.05	166.91	103.12	109.33	100.10	107.99	169.01
Total suitable area	411.20	450.18	423.89	422.57	418.91	423.71	470.19
Percentage of total suitable area	42.83%	46.89%	44.16%	44.02%	43.64%	44.14%	48.98%

The percentage is the ratio of suitable areas to national land surface area (960×10^4^ km^2^) under different climatic scenarios.

**Figure 8:**
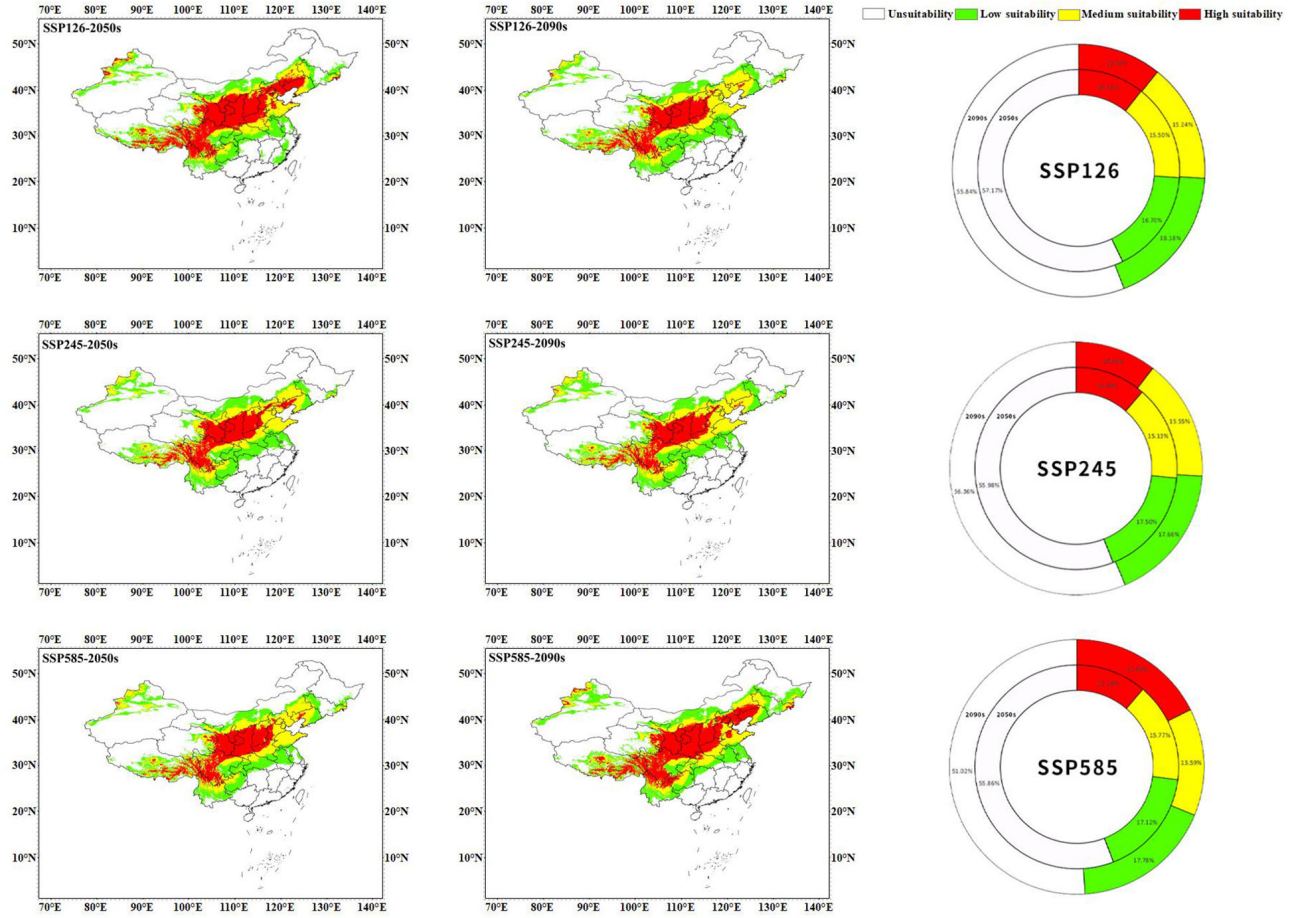
The prediction of suitable areas for *C*. *multiflorus* under future climate scenarios.

From the results of predicting suitable areas for *C*. *multiflorus* under future climate scenarios (**Figure 8**; **Table 3**), it can be seen that in the 2050s, the total suitable area of *C. multiflorus* under SSP126, SSP245, and SSP585 slightly increased. It is worth noting that the highly and medium suitable area of *C. multiflorus* under the background of SSP126 increased by 63.55% and decreased by 26.07%, respectively, compared with that under the current background. The low suitable area increased only by 3.05%, 4.81%, and 2.52%, respectively. In the 2090s, under the different backgrounds of SSP126, SSP245, and SSP585, the total suitable area of *C. multiflorus* also increased. The highly and medium suitable area of *C. multiflorus* under the background of SSP585 increased by 65.61% and decreased by 14.07%, respectively, compared with that under the current background. The low suitable area had no obvious change.

Under the SSP126 climate scenario, the total suitable area in the 2090s decreased by 2.74 percentage points compared to the 2050s. Specifically, the medium and low suitable area increased by 5.62% and 23.90%, respectively, while the highly suitable area decreased by 61.86%. Under the SSP245 climate scenario, there was little difference in the total and different grades of suitable area between the 2050s and 2090s. Under the SSP585 climate scenario, the total suitable area in the 2090s increased by 4.84 percentage points compared to the 2050s. Specifically, the highly and low suitable area increased by 56.50% and 3.87%, respectively, while the medium suitable area decreased by 16.01%.

Overall, the SSP126, SSP245, and SSP585 climate scenarios showed that the total suitable area and highly suitable area in the 2050s and 2090s were larger than the current condition (except for SSP245-2090s). The differences in the medium and low suitable area (165.467×10^4^ km²~174.771×10^4^ km²) between the 2050s and 2090s under different climate scenarios were not significant compared to that under the current condition (160.484×10^4^ km²), except for SSP126-2050s.

### 3.4 Distribution pattern change of *C. multiflorus* in future climate scenarios

**Figure 9:**
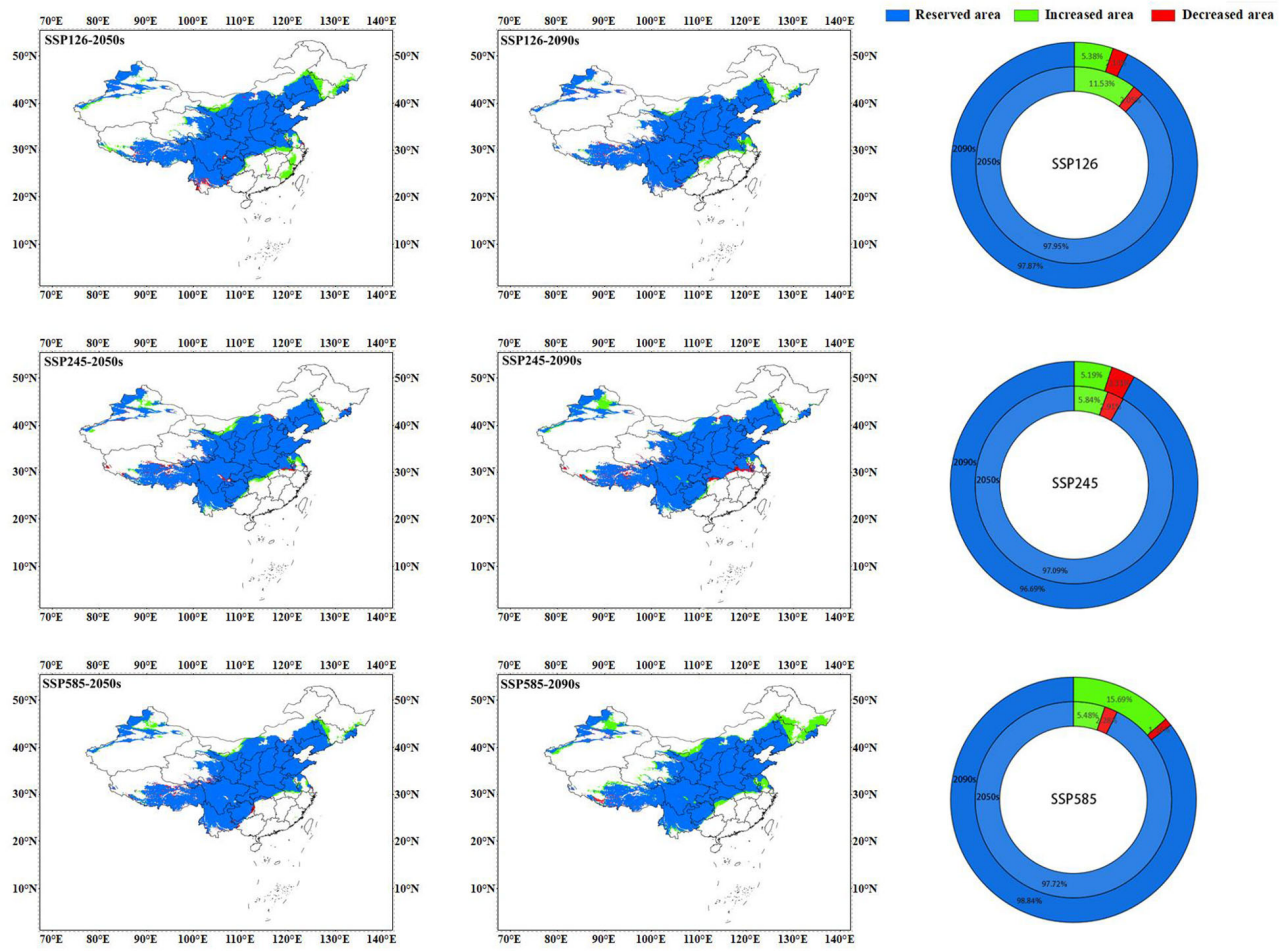
Spatial change of geographical distribution pattern of *C. multiflorus* under future climate scenarios.

The map of spatial change of geographical distribution pattern (**Figure 9**; **Table 4**) showed that the top two climate scenarios with the highest growth rate of potential suitable areas for *C. multiflorus* in the future were SSP126-2050s (11.53%) and SSP585-2090s (15.69%), other future climate scenarios ranged from 5.19% to 5.84%. It is worth noting that under the SSP126-2050s scenario, potential suitable areas of *C. multiflorus* would first appear in Zhejiang, Fujian, and Jiangxi. The top two climate scenarios with the highest loss rate of potential suitable areas were SSP245-2090s (3.31%) and SSP245-2050s (2.91%), but all loss areas were intermittent and small areas. Overall, the retention rates of potential suitable areas for *C. multiflorus* under different future climate scenarios ranged from 96.69% to 98.84%, showing an increasing trend (1.88~14.53%).

**Table 4:** Spatial change of suitable areas for *C. multiflorus* in different periods.

Period	Area (×10^4^ km^2^)				Change Rate (%)			
	Increase	Reserved	Lost	Change	Increase rate	Reserved rate	Lost rate	Change rate
SSP126-2050s	22.147	402.95	8.749	13.397	5.38%	97.87%	2.13%	3.25%
SSP245-2050s	24.046	399.834	12.002	12.044	5.84%	97.09%	2.91%	2.92%
SSP245-2090s	21.361	398.086	13.635	7.726	5.19%	96.69%	3.31%	1.88%
SSP585-2050s	22.545	401.785	9.384	13.161	5.48%	97.72%	2.28%	3.20%
SSP585-2090s	64.532	406.453	4.78	59.752	15.69%	98.84%	1.16%	14.53%

### 3.5 Analysis of niche breadth of main shrub species within *C. multiflorus* community

The importance value is an indicator that describes the significance of a plant species in a community. A higher importance value generally implies that the species holds a crucial position in the community, with a larger population size and wider distribution range compared to other populations. Niche breadth reflects the adaptability of a population within an ecosystem (Cai et al., 2021). From **Table 5**, we can observe that *C. multiflorus* had the highest importance value (10.48) within the shrub community. However, its niche breadth, as measured by the Levins index, ranks third (*B_L_* = 0.59), and fourth according to the Shannon index (*B_S_* =3.16).

**Table 5:** Niche breadth of main tree species in *C*. *multiflorus* community.

NO.	Species	Importance Value (%)	Niche breadth	
			*B_L_*	*B_S_*
1	C. multiflorus	10.48	0.59	3.16
2	*Crataegus kansuensis*	6.39	0.54	3.09
3	*Rosa bella*	5.47	0.78	3.39
4	*Corylus mandshurica*	4.96	0.47	3.12
5	*Rosa bella*	4.25	0.51	3.16
6	*Spiraea pubescens*	3.37	0.58	3.22
7	*Philadelphus incanus*	3.3	0.57	3.12
8	*Ostryopsis davidiana*	3.24	0.21	2.43
9	*Viburnum opulus*	3.17	0.18	2.64
10	*Euonymus phellomanus*	2.98	0.51	3.08
11	*Syringa reticulata*	2.85	0.19	2.37
12	*Cornus macrophylla*	2.73	0.42	2.99
13	*Lonicera ferdinandi*	2.57	0.46	3.12
14	*Quercus mongolica*	2.29	0.19	2.54
15	*Viburnum mongolicum*	2.15	0.61	3.25

### 3.6 Analysis of niche overlap of main shrub species within *C. multiflorus* community

As shown in **Table 6**, within *C. multiflorus* shrub community, 105 species pairs were formed by the 15 major shrub plant species. The niche overlap index (*O_ik_*) ranged from 0.09 to 0.79. Among these pairs, 63 pairs (60% of total pairs) had insignificant overlap (0<*O_ik_* < 0.5) and 42 pairs (40% of total pairs) had significant overlap (*O_ik_* > 0.5), suggesting that the ecological habits of these species pairs were not highly similar. 9 pairs with an *O_ik_* value above 0.7, and most of these pairs consisted of species with relatively wide niches. Among them, *C. multiflorus*-*Philadelphus incanus* had the highest niche overlap (0.79). There were 16 pairs with an *O_ik_* value less than 0.2, and these pairs had relatively narrow niche breadth, such as *Ostryopsis davidiana* - *Viburnum opulus* subsp. *calvescens* (0.09), *O. davidiana* - *Quercus mongolica* (0.09), and *Syringa reticulata* subsp. *amurensis* - *Q. mongolica* (0.09). Overall, the average *O_ik_* value was 0.43, relatively fewer pairs with high overlap.

**Table 6:** Niche overlap of main shrub species in *C. multiflorus* community.

	1	2	3	4	5	6	7	8	9	10	11	12	13	14	15
1	1														
2	0.46	1													
3	0.75	0.67	1												
4	0.4	0.41	0.56	1											
5	0.73	0.46	0.73	0.36	1										
6	0.52	0.57	0.68	0.63	0.59	1									
7	0.79	0.47	0.74	0.45	0.7	0.45	1								
8	0.53	0.33	0.46	0.14	0.39	0.34	0.55	1							
9	0.25	0.37	0.38	0.32	0.3	0.24	0.39	0.09	1						
10	0.61	0.52	0.6	0.51	0.51	0.47	0.5	0.17	0.28	1					
11	0.24	0.34	0.22	0.2	0.15	0.34	0.18	0.19	0.09	0.18	1				
12	0.41	0.48	0.56	0.66	0.45	0.61	0.54	0.14	0.29	0.49	0.18	1			
13	0.44	0.57	0.59	0.42	0.7	0.63	0.46	0.32	0.25	0.37	0.32	0.58	1		
14	0.2	0.2	0.44	0.43	0.31	0.26	0.24	0.09	0.17	0.5	0.09	0.28	0.56	1	
15	0.63	0.59	0.71	0.58	0.63	0.46	0.66	0.31	0.5	0.64	0.21	0.62	0.73	0.35	1

1. *C. multiflorus*; 2. *C. kansuensis*; 3. *R. bella*; 4. *C. mandshurica*; 5. *S. oblata*; 6. *S. pubescens*; 7. *P. incanus*; 8. *O. davidiana*; 9. *V. opulus*; 10. *E. phellomanus*; 11. *S. reticulata*; 12. *C. macrophylla*; 13. *L. ferdinandi*; 14. *Q. mongolica*; 15. *V. mon*.

## 4 Discussion

### 4.1 Model optimization and accuracy evaluation

This study employed the ENMeval 2.0 version, which successfully solved the problems present in previous versions of the MaxEnt model, such as lacking optimal parameter selection (Morales et al., 2017), and overfitting (Merow et al., 2014; Elith and Graham, 2009), which led to inaccurate results. The ENMeval 2.0 version also helps researchers in resolving issues related to insufficient model performance and parameterization reporting, heavy reliance on AICc for model selection, and underutilization of spatial cross-validation (Kass et al., 2021).Therefore, this study employed the ENMeval 2.0 package to determine the optimal parameters for the MaxEnt model, ultimately identifying FC as LQPTH and RM as 2.5. Based on the optimized MaxEnt model, the simulated current distribution range of *C. multiflorus* was roughly consistent with the records in the Flora of China. Moreover, the AUC value (0.875) exceeded 0.8, indicating a high level of credibility for this simulation and providing a research basis for subsequent investigations (Araújo et al., 2005; Tang et al., 2021; Mahmoodi et al., 2022). The result is also useful to the collections of *C. multiflorus* germplasm resources as well as seed introduction.

### 4.2 Constraints of environmental variables on the potential distribution of *C. multiflorus*

Jackknife test (**Figure 5**) revealed that the top three variables significantly influencing the distribution of *C. multiflorus* were Annual precipitation (Bio12), Min air temperature of the coldest month (Bio6), and Mean air temperature of the coldest quarter (Bio11), which aligned with the environmental factors affecting the distribution of *Haloxylon*, a genus with drought-resistance (Zhang et al., 2022). Among these, the contribution rate and the importance value of Annual precipitation (Bio12) were the highest, indicating that in arid and semi-arid regions, precipitation is more important than temperature. This is consistent with the findings of Qin et al. (2023), who observed that extreme drought in certain areas consumed soil moisture reserves, restricting plant survival and growth. Furthermore, when Annual precipitation (Bio12) exceeded 150 mm, *C. multiflorus* can survive, and when it exceeded 500 mm, the survival rate was 0.60 (**Figure 6**). This further indicates that *C. multiflorus* exhibits strong tolerance to drought and may serve as a pioneer species for ecological restoration in arid and semi-arid areas.

Climate factors can not completely determine the geographical distribution of *C. multiflorus*. Soil conditions, human activities, and other factors also play important roles in shaping the distribution of plants at different spatial scales (Shishir et al., 2020; Xu W. B. et al., 2019). For instance, the seeds of *C. multiflorus* have hard outer shells, waxy inner membranes, poor permeability, and long physiological dormancy periods (Yu et al., 2017). Some scholars found that seeds not subjected to cold storage will not germinate the following year (Chi et al., 2015). These seed properties may influence the growth and distribution of *C. multiflorus*, consistent with the findings of Zhou et al. (2016) and Alzate et al. (2023). Additionally, it reproduces both sexually (inter-species hybridization) and asexually (Nybom and Bartish, 2007; Meng et al., 2021). This not only increases its genetic diversity and stress resistance to some extent but also effectively stabilizes hybrid advantages (Patel et al., 2018; Nitta et al., 2022), making it possible to adapt to various extreme climates. Therefore, in order to predict the distribution of plants accurately, it is necessary to consider more environmental factors and bio-ecological characteristics.

### 4.3 The potential geographical range of *C. multiflorus* under future climate change scenarios

With the exponential growth of global greenhouse gas emissions, the trend of future climate warming in China will be further exacerbated (Li et al., 2020). *C. multiflorus* will be able to effectively respond to global warming. Under different future climate scenarios, the proportion of the total suitable area for *C. multiflorus* ranges from 43.64% to 48.98%, which is 0.8 to 6.14 percentage points higher than the current situation. This suggests that, within a certain range, moderate climate warming may facilitate species dispersal and population expansion (Bazzaz, 1990; Thomas et al., 2004; Schöb et al., 2009), especially benefiting drought-resistant shrub species (Screen, 2014).

Previous studies have shown that the climate scenario SSP245 is closest to the actual predicted climate conditions in China (Gao et al., 2013; Lai et al., 2023). However, under the climate scenario SSP126-2050s with the lowest carbon emissions, the total and highly suitable area of *C. multiflorus* increased by 4.06 and 11.53 percentage points compared to that of the current climate scenario, respectively. In addition to expanding to the high latitude areas of Liaoning in northeastern China and the southern part of Heilongjiang, *C. multiflorus* also expanded to the lower latitude regions of Anhui, and appeared for the first time in Zhejiang, Fujian, and some parts of Jiangxi. However, with the continuous increase in greenhouse gas emissions (SSP126-2090s), *C. multiflorus* disappeared in the above areas. This indicated that under low carbon emissions, these areas could meet the most suitable growth condition for *C. multiflorus*, but their advantages might disappeared when the climate turned warm.

**Figure 10:**
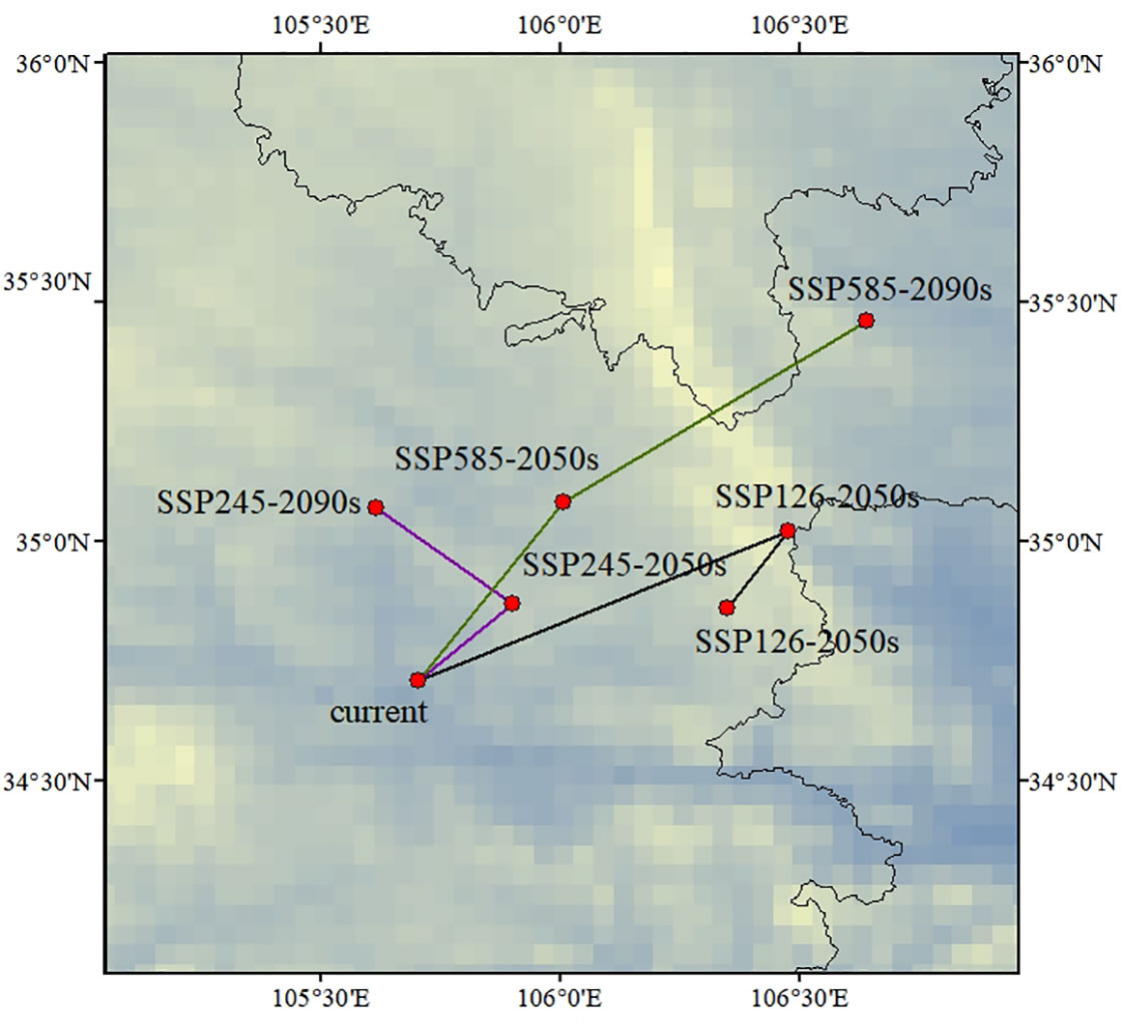
Migration location of the center of suitable areas for *C. multiflorus* during periods.

Under all the climate scenarios, *C. multiflorus* expanded towards higher latitudes (**Figure 10**), mainly towards the northern part of Xinjiang, Liaoning, and the southern part of Heilongjiang in northeastern China. This might be due to the fact that with the emission of greenhouse gases, the rainfall would gradually increase from the current altitude to higher altitudes (Jiang et al., 2022), while the temperature in temperate continental and cold temperate arid climate regions would also rise, which was more conducive to the growth of *C. multiflorus*. This is consistent with previous studies, which indicate that with the intensification of global warming, the distribution of most species will shift to higher latitudes and altitudes (Zhang et al., 2018; Faticov et al., 2021).

### 4.4 The niche characteristics within *C. multiflorus* community

This study used MaxEnt model and CMIP6 data to simulate the potential suitable areas of *C. multiflorus* under different climate change scenarios. However, its distribution is not only affected by climatic factors, but also by population distribution (Theunis et al., 2005), community structure (Zhao et al., 2022) and many other factors. Therefore, when planning forest management and formulating restoration strategies, more attention should be paid to the differences of niche between species, which are crucial for explaining species coexistence at regional scales, as well as sustainable conservation and management of forests under climate change.

Niche analysis helps to understand coexistence mechanisms, predict community responses (Huang et al., 2019), and explain environmental tolerance of species (Smith et al., 2019). As shown in **Tables 5**, **6**, the shrub community mainly consisted of 10 tree species from the Rosaceae, Betulaceae, Oleaceae, Hydrangeaceae, Adoxaceae, Celastraceae, Cornaceae, Caprifoliaceae, and Fagaceae families, indicating high species diversity and strong structural stability. *C. multiflorus* had the highest importance value (10.48), and could coexist with other tree species, demonstrating strong adaptability and dominance in community.

Interestingly, the presence of two woody tree species, *Cornus macrophylla* and *Q. mongolica*, within the shrub community suggested that the *C. multiflorus* community could adapt to relatively harsh conditions and begin to accumulate soil moisture and nutrients, promoting the construction of forest communities. The niche breadth of *C. multiflorus* ranked third, and the Shannon index ranked fourth with significant overlap with species such as *Rosa bella*, *Syringa oblata*, *P. incanus*, *Euonymus phellomanus*, *Viburnum mongolicum*, *O. davidiana*, and *Spiraea ouensanensis*, suggesting that these species may have a competitive relationship with *C. multiflorus*. Therefore, in ecologically fragile environments, it is advisable to first establish a *C. multiflorus* community, and avoid species with significantly overlapping niches, such as *R. bella*, *S. oblata*, *P. incanus*, *E. phellomanus*, *V. mongolicum*, *O. davidiana*, and *S. ouensanensis*.

## 5 Conclusion

In this study, we predicted the potential spatial pattern of *C. multiflorus* in China by MaxEnt 2.0 model, combined with climatic factors and field investigation. Besides, we explored the main climatic factors and ecological adaptability which affect its distribution. The results showed that the main climatic factors were Annual precipitation (Bio12), Min air temperature of the coldest month (Bio6) and Mean air temperature of the coldest quarter (Bio11). *C. multiflorus* can grow in the environment with Annual precipitation (Bio12) >150mm, Min air temperature of the coldest month (Bio6) > -42.5°C and Mean air temperature of the coldest quarter (Bio11) > -20°C, which is in line with the characteristics of high cold and drought resistance. In addition, its distribution will further expand to higher latitudes. *C. multiflorus* can show dominance in communities, while coexists with other shrub species. In conclusion, under the trend of global warming, *C. multiflorus* can be regarded as a pioneer species for ecological restoration in arid and semi-arid areas.

## Data availability statement

The original contributions presented in the study are included in the article/**Supplementary Material**. Further inquiries can be directed to the corresponding author.

## Author contributions

QH: Conceptualization, Data curation, Formal Analysis, Methodology, Validation, Visualization, Writing – original draft, Writing – review & editing. HL: Writing – original draft, Validation. CL: Data curation, Formal Analysis, Writing – original draft. XZ: Investigation, Writing – original draft, Data curation. ZY: Formal Analysis, Writing – original draft. JL: Data curation, Investigation, Visualization, Writing – original draft. MC: Data curation, Investigation, Writing – original draft. ZH: Data curation, Investigation, Writing – original draft. YY: Data curation, Writing – original draft. SZ: Data curation, Writing – original draft. ZL: Data curation, Writing – original draft. GZ: Conceptualization, Funding acquisition, Project administration, Supervision, Visualization, Writing – review & editing.

## Footnotes

1
www.gbif.org

2
http://www.nsii.org.cn/

3
http://www.cvh.ac.cn/

4
http://www.iplant.cn/
